# Use of Tumor Markers in Gastrointestinal Cancers: Surgeon Perceptions and Cost-Benefit Trade-Off Analysis

**DOI:** 10.1245/s10434-016-5717-y

**Published:** 2016-12-22

**Authors:** Amish Acharya, Sheraz R. Markar, Michael Matar, Melody Ni, George B. Hanna

**Affiliations:** Division of Surgery, Department of Surgery and Cancer, St Mary’s Hospital, Imperial College London, 10th Floor QEQM Building, South Wharf Road, London, W2 1NY UK

## Abstract

**Background:**

Gastrointestinal cancers constitute the third most common cancers worldwide. Tumor markers have long since been used in the postoperative surveillance of these malignancies; however, the true value in clinical practice remains undetermined.

**Objective:**

This study aimed to evaluate the clinical utility of three tumor markers in colorectal and esophagogastric cancer.

**Methods:**

A systematic review of the literature was undertaken to elicit the sensitivity, specificity, statistical heterogeneity and ability to predict recurrence and metastases for carcinoembryonic antigen (CEA), cancer antigen (CA) 19-9 and CA125. European surgeons were surveyed to assess their current practice and the characteristics of tumor markers they most valued. Data from the included studies and survey were combined in a cost-benefit trade-off analysis to assess which tumor markers are of most use in clinical practice.

**Results:**

Diagnostic sensitivity and specificity were ranked the most desirable characteristics of a tumor marker by those surveyed. Overall, 156 studies were included to inform the cost-benefit trade-off. The cost-benefit trade-off showed that CEA outperformed both CA19-9 and CA125, with lower financial cost and a higher sensitivity, and diagnostic accuracy for metastases at presentation (area under the curve [AUC] 0.70 vs. 0.61 vs. 0.46), as well as similar diagnostic accuracy for recurrence (AUC 0.46 vs. 0.48).

**Conclusions:**

Cost-benefit trade-off analysis identified CEA to be the best performing tumor marker. Further studies should seek to evaluate new tumor markers, with investigation tailored to factors that meet the requirements of practicing clinicians.

**Electronic supplementary material:**

The online version of this article (doi:10.1245/s10434-016-5717-y) contains supplementary material, which is available to authorized users.

Gastrointestinal cancers are the third most common cancers worldwide, with a prevalence of 1,281,539 in the US in 2013.^1^ Survival for gastrointestinal malignancies have been improving worldwide due to advances in multimodality treatments, diagnostic strategies and expanding the criteria for treatable disease. While these strategies commonly involve a combination of radiological and endoscopic techniques, some studies have shown serum tumor markers may have a diagnostic, as well as therapeutic, monitoring role.^2^,^3^

A tumor marker is defined as a compound produced by the tumor or the host, in response to a malignancy. Traditionally, markers such as carcinoembryonic antigen (CEA) and, to a lesser extent, cancer antigen (CA) 19-9, have been used clinically to monitor disease response, whereby the efficacy of treatments can be assessed by noting a reduction in the level of a marker, which was previously high.^4^ In colorectal cancer, the use of tumor makers in postoperative surveillance has been recommended by the American Society of Clinical Oncology; however, their use in the identification of metastasis and diagnostic accuracy has not been established^5^ despite some clinicians incorporating them into regular practice. In contrast, the use of tumor markers in esophagogastric malignancies in any capacity remains controversial. A number of studies have assessed the use of several tumor markers in prognosis and diagnosis, however they are of limited quality.^6^,^7^ As such, there is no clear consensus on the use of tumor markers, and current practice is dependent on the individual clinician’s choice.

In both cancer types, there is hence a clear need for an objective evaluation of common tumor markers in several clinical scenarios. This appraisal requires a comparison of clinical utility and costs or negatives with the utilization of the marker; however the relative importance of these benefits and costs, or performance characteristics, as drivers to uptake has not yet been quantified by the literature and would be dependent on how clinicians perceive the use of tumor markers. This study aimed to critically assess the cost-benefit trade-off of three common tumor markers in gastrointestinal cancers by means of evaluating the perceptions of surgeons to common markers.

## Methods

### Literature Search Strategy

A literature search of the PubMed, Ovid MEDLINE, EMBASE and Google Scholar electronic databases was conducted from January 1990 up to and including December 2015 for studies regarding the use of tumor markers in the diagnosis, postoperative surveillance, or prediction of metastasis in colorectal and esophagogastric cancer (Online Appendix 1). Search terms used included ‘colorectal neoplasms’, ‘esophageal cancer’, ‘gastric cancer’, ‘tumour markers’, ‘neoplasm antigens’, ‘tumour-associated antigens’, ‘prognosis’, ‘recurrence’, ‘metastasis’ and ‘staging’ in various combinations, as well as the name of the specific markers, relevant surgical procedures, and alternative spellings, e.g. tumor.

Research titles were then screened for suitability, with full-text copies retrieved. All studies that investigated the diagnostic, prognostic, or predictive ability of a single or multiple tumor marker in colorectal or esophagogastric cancers that could be tested in patients were included. Exclusion criteria involved studies with no available English translation, published abstracts only, and those assessing the predictive ability for metastases in which no diagnostic accuracy or recurrence data could be calculated.

Of those studies meeting the inclusion criteria, the stated specificity and sensitivity were extracted. Studies that did not explicitly state the sensitivity and specificity of the marker were independently calculated and verified by two authors (AA and SRM), provided sufficient data were available.

### Literature Standard

The QUADAS-2^8^ tool, which involves four domains, i.e. patient selection, index test, reference standard, and flow of subjects through the study, was used to appraise the standard of the literature, and was implemented to assess the quality and risk of bias of the included studies. The reference standard was histological confirmation of malignancy or recurrence.

### Tumor Marker Survey

Surgeons affiliated with the European Association of Endoscopic Surgery were invited to complete an anonymous survey regarding tumor markers. These surgeons were asked to rank attributes of the ‘ideal marker’ in order of their perceived importance to routine clinical practice (Online Appendix 2). No duplication of rank was permitted. These characteristics included diagnostic sensitivity, specificity, consistency across demographics, patient acceptability, cost, time for result, and predictive of recurrence and metastases (defined by the AUC). A summative rank was then calculated and informed the weighting for the cost-benefit trade-off analysis.

### Statistical Methodology

For each of the assessments of cancer diagnosis, recurrence, and metastasis, paired sensitivity and specificity were calculated from each eligible study, as appropriate. A bivariate model for meta-analysis of statistical accuracy provides more accurate results than fixed-effects modeling. Following the validated methodology of Harbord et al.^9^, bivariate meta-analyses were performed to generate pooled point estimates and 95% confidence intervals (CIs) for the sensitivity and specificity of the tumor marker under investigation, with histopathological confirmation of malignancy, together with hierarchical summary receiver operating characteristic (ROC) curves. The software used for this analysis was the custom-designed statistical package Michigan Interactive Data Analysis System (MIDAS).^10^ Areas under the hierarchical summary ROC curves, as well as I^2^ statistics, were obtained directly from the MIDAS output (see Zhou and Tu for an in-depth description of the statistical methods used.^11^)

### Performance Characteristics

The performance of the three tumor markers, with respect to the eight characteristics surgeons were asked to rank in the survey, was calculated (Tables 1, 2). Cost and speed of result were taken as the stated laboratory process costs from St Mary’s Hospital, Paddington, UK. Sensitivity, specificity, prediction of metastases at primary diagnosis, and recurrence following resection were calculated from the aforementioned pooled analyses, with the latter two represented by the area under the curve (AUC). Consistency was calculated from the I^2^ heterogeneity statistic from the included studies, representing the percentage of total variation across the studies, with a higher number meaning lower consistency.

**Table 1:** Performance of CEA, CA19-9 and CA125 with respect to the eight performance characteristics for colorectal cancer

Characteristic desirability from the survey	Characteristic	CEA	CA19-9	CA125
1	Diagnostic sensitivity	0.53	0.47	0.20
2	Diagnostic specificity	0.86	0.92	0.99
3	Predictive of recurrence	0.78	0.55	0.55
4	Predictive of metastasis	0.77	0.00	0.00
5	Low cost (£)	8.00	16.00	17.00
6	Speed of result (hours)	24.00	24.00	24.00
7	Patient acceptability	Good	Good	Good
8	Consistency	0.86	0.96	0.96

*CEA* carcinoembryonic antigen, *CA* cancer antigen

**Table 2:** Performance of CEA, CA19-9 and CA125 with respect to the eight performance characteristics for esophagogastric cancer

Characteristic desirability from the survey	Characteristic	CEA	CA19-9	CA125
1	Diagnostic sensitivity	0.34	0.30	0.31
2	Diagnostic specificity	0.85	0.81	0.94
3	Predictive of recurrence	0.40	0.48	0.00
4	Predictive of metastasis	0.70	0.61	0.46
5	Low cost (£)	8.00	16.00	17.00
6	Speed of result (hours)	24.00	24.00	24.00
7	Patient acceptability	Good	Good	Good
8	Consistency	0.93	0.98	0.96

*CEA* carcinoembryonic antigen, *CA* cancer antigen

### Cost-Benefit Trade-Off Analysis

The eight performance characteristics were broadly divided into either costs (time for result and financial cost) or benefits (sensitivity, specificity, predictive ability for recurrence or metastases and consistency). To assess trade-offs between costs and benefits among the tumor markers, we employed Multi-criteria decision analysis (MCDA) methods.^12^–^15^ To achieve this, we rated each tumor marker on all the performance characteristics (*criteria ratings*), and assessed the relative importance of performance characteristics (*criteria weights*) based on the average rankings retrieved from the tumor marker survey (see above). Using a weighted average model, we then combined the ratings to produce an *overall benefit score* for each tumor marker, and then contrasted the benefit scores against the scores on costs and time criteria, respectively.

### Criteria Ratings

Within MCDA, the performance of each tumor marker (so-called a criteria rating), with respect to each characteristic, is bounded between 0, assigned to the worst-performing tumor marker (e.g. most expensive or least sensitive), and 100 for the best-performing marker (e.g. cheapest or most sensitive). We assumed linearity between performance and rating, using linear interpolation to assess criteria ratings of any intermediate performance. For instance, if the costs of one tumor marker were halfway between the most expensive and the least expensive options, then this tumor marker received a criteria rating of 50 with respect to *cost*.

### Criteria Weights

Trade-offs between the criteria are achieved through ‘criteria weights’, which capture the relative importance of the eight performance characteristics. From the tumor marker survey, respondents provided rankings as to the desirability of each of the characteristics. We converted the average rankings into numerical weights by assigning a criteria weight of 100 to the highest ranked performance characteristic, 90 to the second highest ranked, and so forth. We then normalized all the weightings so that they totaled 1. For example, if sensitivity received a criteria weight of 100, whereas specificity received a weight of 50, this would imply that the surgeons considered a difference of 10%, with respect to the diagnostic sensitivity between two tumor markers, to be equivocal to a difference of 20% in their relative diagnostic specificities.

### Overall Benefit Scores

Our aim was to assess trade-offs between the costs and benefits of using a tumor marker. We therefore combined the ratings of the characteristics previously designated as *benefits* into an overall score. Under the assumption that these characteristics are independent of each other, we can assess the overall score by a weighted average model:^14^

$${\text{Benefit}} = \sum_{k} W_{k} R_{k}$$

where *R*_*k*_ is the rating on the *k*th benefit criterion and *W*_*k*_ is the weight assigned to that criterion.

For instance, suppose the benefit of tumor markers comprises their sensitivity and specificity alone. Assuming that a tumor marker receives ratings of 100 and 50 on sensitivity and specificity, respectively, which have normalized weights of 0.75 and 0.25, it follows that the benefit score of this tumor marker is 87.5 (=100 × 0.75 + 50 + 0.25).

### Trade-Offs

Using normalized weights, the benefit scores are bounded between 0 and 100; a hypothetical tumor marker that has the worst performances on any criteria is scored 0, whereas a tumor marker that has the best performances is scored 100. The higher the score, the more beneficial the tumor marker is and vice versa. We plotted two-way cost-benefit maps to compare benefit against financial costs, and separately from time to results. The analysis was performed using the decision analytic software *HiView,* version 3.2.0.7 (educational copy).

## Results

### Literature Search

A total of 151 full-text articles met the inclusion criteria and were appraised following the literature search (Online Appendix 2), constituting a total of 39,857 patients (19, 634 with colorectal malignancy and 20, 223 with esophagogastric malignancy). Overall, 102 articles assessed colorectal tumor markers (42 on diagnostic ability, 28 on prediction of recurrence, and 32 on prediction of metastases), and 112 articles assessed esophagogastric tumor markers (71 on diagnostic ability, 15 on prediction of recurrence, and 26 on prediction of metastases).

### QUADAS-2 Evaluation

**Fig. 1:**
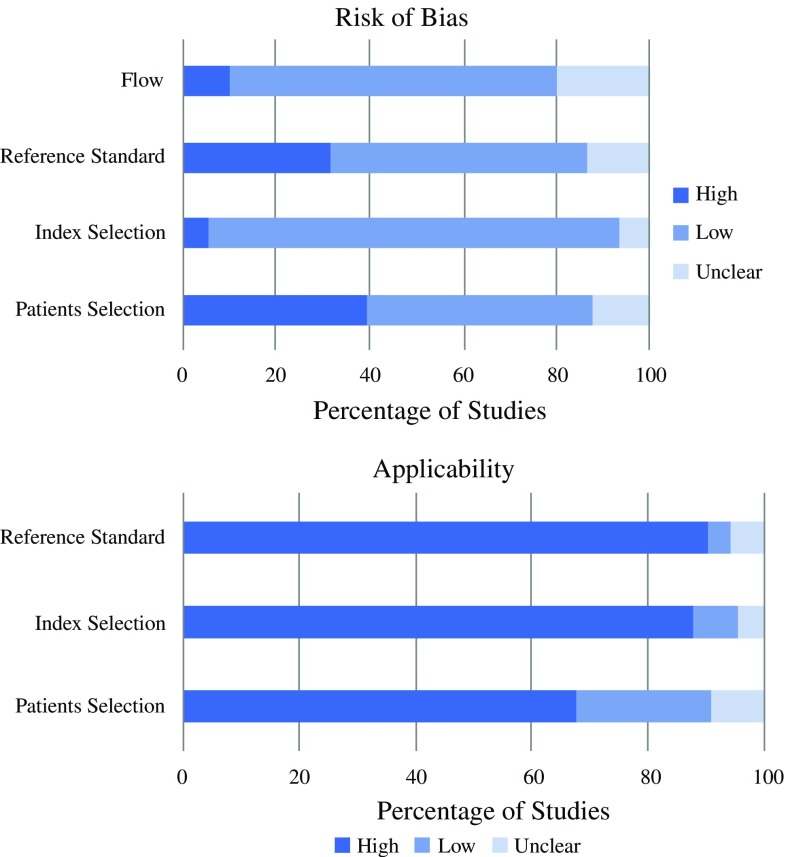
Results of the QUADAS-2 evaluation of the included studies with respect to the risk of bias and applicability

The results of the QUADAS-2 evaluation are shown in Fig. 1. Of the 151 studies included, 40% had a ‘high risk’ of bias with respect to patient selection, while a further 12% did not provide sufficient detail on exclusion criteria for patients enrolled in the study. With respect to the reference standard, 32% had a ‘high risk’ of bias due to their retrospective nature.

### Pooled Analysis for Diagnostic Sensitivity and Specificity

The pooled diagnostic sensitivity and specificity of CEA in colorectal cancer was 0.53 (95% CI 0.50–0.56) and 0.86 (95% CI 0.85–0.88), respectively (Table 3). For CA19-9, sensitivity was 0.47 (95% CI 0.44–0.51) and specificity was 0.92 (95% CI 0.91–0.94) and, for CA125, pooled diagnostic sensitivity was 0.20 (95% CI 0.15–0.26) and specificity was 0.99 (95% CI 0.98–1.00). Heterogeneity for diagnosis determined using the I^2^ statistic, which represented the consistency, was 0.86 for CEA and 0.96 for CA19-9 and CA125. Similar results were found for esophagogastric cancer (Table 4).

**Table 3:** Results of the pooled sensitivity, specificity and AUC from the literature search for diagnostic ability, predictive ability of recurrence and metastases for CEA, CA19-9 and CA125, with respect to colorectal cancer

Tumor marker	Diagnosis			Diagnosis of recurrence			Diagnosis of metastases		
	Sensitivity	Specificity	AUC	Sensitivity	Specificity	AUC	Sensitivity	Specificity	AUC
CEA	0.533 (0.502–0.563)	0.864 (0.848–0.881)	0.704	0.550 (0.527–0.571)	0.808 (0.796–0.820)	0.779	0.632 (0.600–0.664)	0.771 (0.753–0.789)	0.771
CA19-9	0.471 (0.436–0.506)	0.924 (0.907–0.939)	0.674	0.325 (0.289–0.363)	0.751 (0.721–0.789	0.549	0.000*	0.000*	0.000*
CA125	0.198 (0.148–0.257)	0.993 (0.977–0.999)	0.993	0.245 (0.198–0.287)	0.639 (0.593–0.683)	0.550	0.000*	0.000*	0.000*

Values in parentheses represent 95% confidence intervals

*AUC* area under the curve, *CEA* carcinoembryonic antigen, *CA* cancer antigen

* Insufficient data available

**Table 4:** Results of the pooled sensitivity, specificity and AUC from the literature search for diagnostic ability, predictive ability of recurrence and metastases for CEA, CA19-9 and CA125, with respect to esophagogastric cancer

Tumor marker	Diagnosis			Prediction of recurrence			Prediction of metastases		
	Sensitivity	Specificity	AUC	Sensitivity	Specificity	AUC	Sensitivity	Specificity	AUC
CEA	0.343 (0.322–0.365)	0.853 (0.840–0.866)	0.523	0.335 (0.303–0.368)	0.860 (0.848–0.872)	0.464	0.241 (0.221–0.257)	0.241 (0.221–0.257)	0.699
CA19-9	0.298 (0.278–0.318)	0.812 (0.791–0.831)	0.663	0.258 (0.225–0.299)	0.854 (0.839–0.868)	0.478	0.195 (0.80–0.210)	0.195 (0.180–0.210)	0.606
CA125	0.313 (0.264–0.365)	0.935 (0.910–0.954)	0.749	0.000*	0.000*	0.000*	0.332 (0.285–0.381)	0.913 (0.895–0.929)	0.460

Values in parentheses represent 95% confidence intervals

*AUC* area under the curve, *CEA* carcinoembryonic antigen, *CA* cancer antigen

* Insufficient data available

### Tumor Marker Survey

**Fig. 2:**
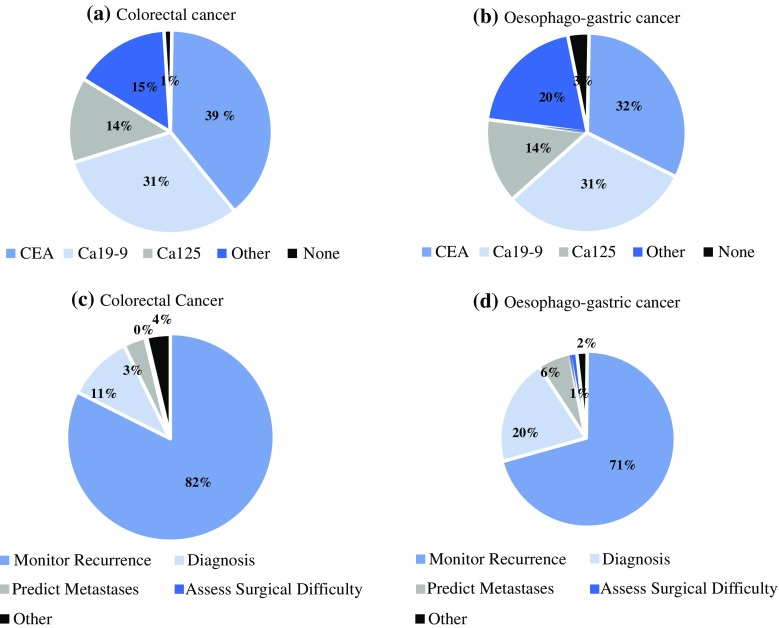
Survey results detailing the most commonly used tumor markers and the most commonly cited indications in (**a, c**) colorectal cancer and (**b, d**) esophagogastric cancer. *CEA* carcinoembryonic antigen, *CA* cancer antigen

The survey was distributed online from 1 August to 1 September 2015, and a total of 443 responses were collected, representing a response rate of 8.1%. There were 273 respondents with an interest in colorectal disease (200 consultants, 59 registrars, and 14 who were primarily academic), and who had completed a median of more than 100 cancer operations. CEA was the most commonly utilized tumor marker in colorectal cancer (Fig. 2a), with surveillance for recurrence the most common indication (Fig. 2c).

With respect to upper gastrointestinal surgery, 170 respondents (131 consultants, 29 registrars, and 10 primarily academic) had completed a median of more than 100 cancer operations. CEA was the most commonly employed tumor marker (Fig. 2b), with assessment for recurrence being the most common indication (Fig. 2d).

Of the eight performance characteristics, the ideal tumor marker would have, diagnostic sensitivity ranked the overall highest (most desirable), followed by diagnostic specificity. Consistency across demographics was considered the least desirable (Tables 1, 2).

### Cost-Benefit Trade-Off

All tumor markers had identical performances with regard to patient acceptability and speed for result. As all were derived from serum, with a result returned within 24 h, these characteristics were removed from the analysis. Tables 1 and 2 display the performance of each tumor marker with respect to the eight characteristics and the associated importance ranking derived from the survey.

**Fig. 3:**
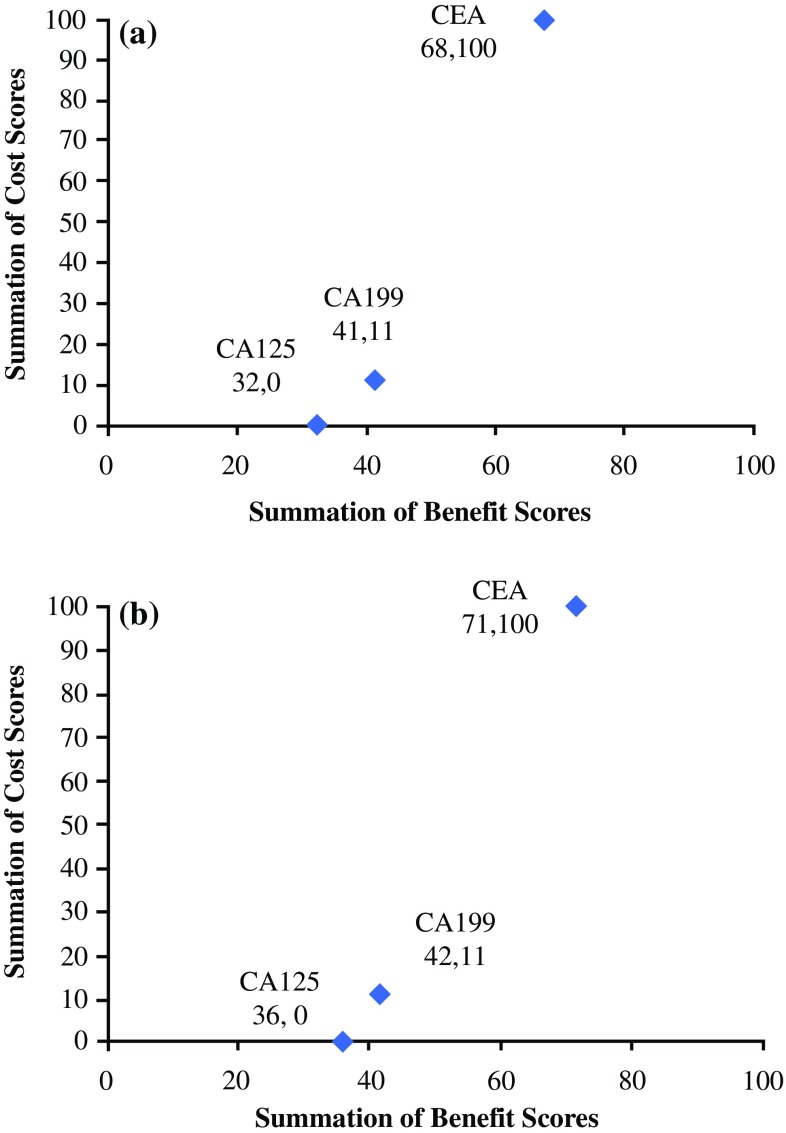
Cost-benefit trade-off for use of the tumor markers in (**a**) colorectal cancer and (**b**) esophagogastric cancer. Higher summated cost score represents lower costs, while higher summated benefit score represents higher benefits. *CEA* carcinoembryonic antigen, *CA* cancer antigen

Figure 3 displays the trade-offs between the benefits (high diagnostic sensitivity, specificity, consistency, predictive ability for recurrence and metastases) and costs (financial). CEA outperformed both CA19-9 and CA125 with respect to overall utility; it had lower associated financial costs and higher benefits, as weighted by the importance placed on the characteristics. This pattern was seen with both colorectal and esophagogastric cancer.

## Discussion

The present study has highlighted the variable performance of common tumor markers in the assessment of gastrointestinal cancers. Despite this variability, the majority of surgeons who were surveyed utilize tumor markers in their practice. CEA was found to have high utility, with a high ability to predict recurrences and metastases, and was also associated with the lowest costs, primarily financial, as all three tumor markers took the same timeframe to attain the result. Therefore, CEA outperformed CA19-9 and CA125 in cost-benefit trade-off with respect to both colorectal and esophagogastric cancer. However, all markers had poor sensitivity, which would suggest their use in diagnosis is significantly limited.^16^,^17^

CEA is a glycoprotein produced in minimal amounts after fetal development, and which is involved in cell adhesion.^18^ The use of CEA in postoperative surveillance is well established, with the majority of surgeons surveyed using tumor markers for monitoring recurrence. We have shown that CEA could be of use in the assessment of metastasis, having a predictive ability of 77%, due to its association with the spread of cancer and increasing tumor burden.

However, the use of CEA in diagnosis is not widely endorsed. Its use is confounded by its association with smokers, and the need for repeated measurements to mitigate limited sensitivity. Despite this, our survey showed 11% of surgeons still utilize tumor markers for diagnosis, whereas we have shown CEA performs similarly to chance, with a diagnostic sensitivity of 50%. Moreover, high diagnostic sensitivity and specificity were the two most desired characteristics of the ‘ideal tumor marker’, despite monitoring of recurrence being the primary role for CEA. Hence, there is a clinical need for an improved diagnostic tumor marker for colorectal cancer. While utilizing a single diagnostic marker is challenging, a diagnostic tumor marker that could be used in conjunction, or even triage further more specific testing, would be of use. Tumor markers are most commonly used in clinical practice in combination rather than in isolation; however, it was not possible to test the combination of tumor markers in this current study due to limitations in the data. None of the traditional tumor markers would satisfy the criteria for use alone clinically, and, as such, research should focus on novel markers with diagnostic ability.^19^,^20^

In contrast to colorectal cancer, there remains no consensus for the use of tumor markers in esophagogastric cancer. Despite this, they appear widely used in clinical practice, with CEA and CA19-9 the most often employed, and monitoring for recurrence the most common indication, but, in as many as 20% of cases, surgeons admitted to using tumor markers for diagnostic investigation. While CEA again outperformed CA19-9 and CA125 overall, all three tumor markers were found to have low diagnostic accuracy, as determined by the AUC. Unlike in colorectal cancer, all three were also found to have a low capacity to predict recurrence, which should preclude their use in widespread clinical practice.

Discrepancy with the use of tumor markers in esophagogastric cancer would suggest further prospective evaluation is warranted, with a degree of discrimination with respect to their clinical interpretation. Furthermore, given the sensitivity of upper gastrointestinal endoscopy of 95%, the capacity for histological evaluation, and surveillance for Barrett’s esophagus,^21^ the pragmatic value of traditional tumor markers for esophagogastric cancer is restricted.

This study has also highlighted the importance of a holistic evaluation of tumor markers prior to incorporating them into clinical practice. While ‘benefits’ such as diagnostic sensitivity and specificity are key characteristics, as seen by their relative importance denoted in the survey, there must be an appreciation of costs. A marker that has high sensitivity but has a high financial cost or requires excessive processing time may be practically precluded from widespread use, which may explain the fact that despite poor diagnostic accuracy and only relatively high predictive ability, the tumor markers this study has appraised are still in widespread use. While novel markers may offer greater diagnostic use, they are also likely to require more esoteric, and therefore expensive, assays.

The limitations of this study are as a result of the published studies included to inform the cost-benefit trade-off. QUADAS evaluation revealed a ‘high’ level of bias in 32% of the studies due to their retrospective design, limiting the reliability of the results obtained from the pooled analyses. Moreover, the potential for bias in some of these studies was high due to their case-control design and restrictive exclusion criteria, as was seen by the ‘high’ risk of bias with respect to patient selection.

As only a few studies assessed the use of CA19-9 and CA125 for the prediction of recurrence or metastases, there were insufficient data to undertake the analyses, and an assumption was therefore required, with the marker scoring zero for that performance characteristic. This would lead to CEA spuriously appearing to have higher benefits, simply as a product of it being more extensively investigated. The trade-off analysis is also informed by the survey, which would only represent the views of members of the European society who responded. This suggests some positive selection bias, especially given the response rate of approximately 8%.

## Conclusions

Tumor markers have long been utilized in the monitoring of gastrointestinal cancers, with variable success. While traditional markers have a use in colorectal cancer surveillance, their use in esophagogastric malignancies is somewhat less defined and requires clarification. In both cases, there appears to be a need for a tumor marker with higher diagnostic accuracy. This would suggest that further areas of research should focus on the search for new novel biomarkers for diagnosis and therapeutic monitoring (See Online Appendix 3 for References of included papers).

## Electronic supplementary material

Below is the link to the electronic supplementary material.

Supplementary material 1 (PDF 181 kb)

Supplementary material 2 (PDF 226 kb)

Supplementary material 3 (PDF 387 kb)
