# Supplementary figures and images for: Use of Tumor Markers in Gastrointestinal Cancers: Surgeon Perceptions and Cost-Benefit Trade-Off Analysis
Source: Ann Surg Oncol. 2016 Dec 22;24(5):1165–73. doi: 10.1245/s10434-016-5717-y (PMC5374165; doi:10.1245/s10434-016-5717-y)

## APPENDICES

**Appendix 1:** Diagram detailing the literature search strategy.

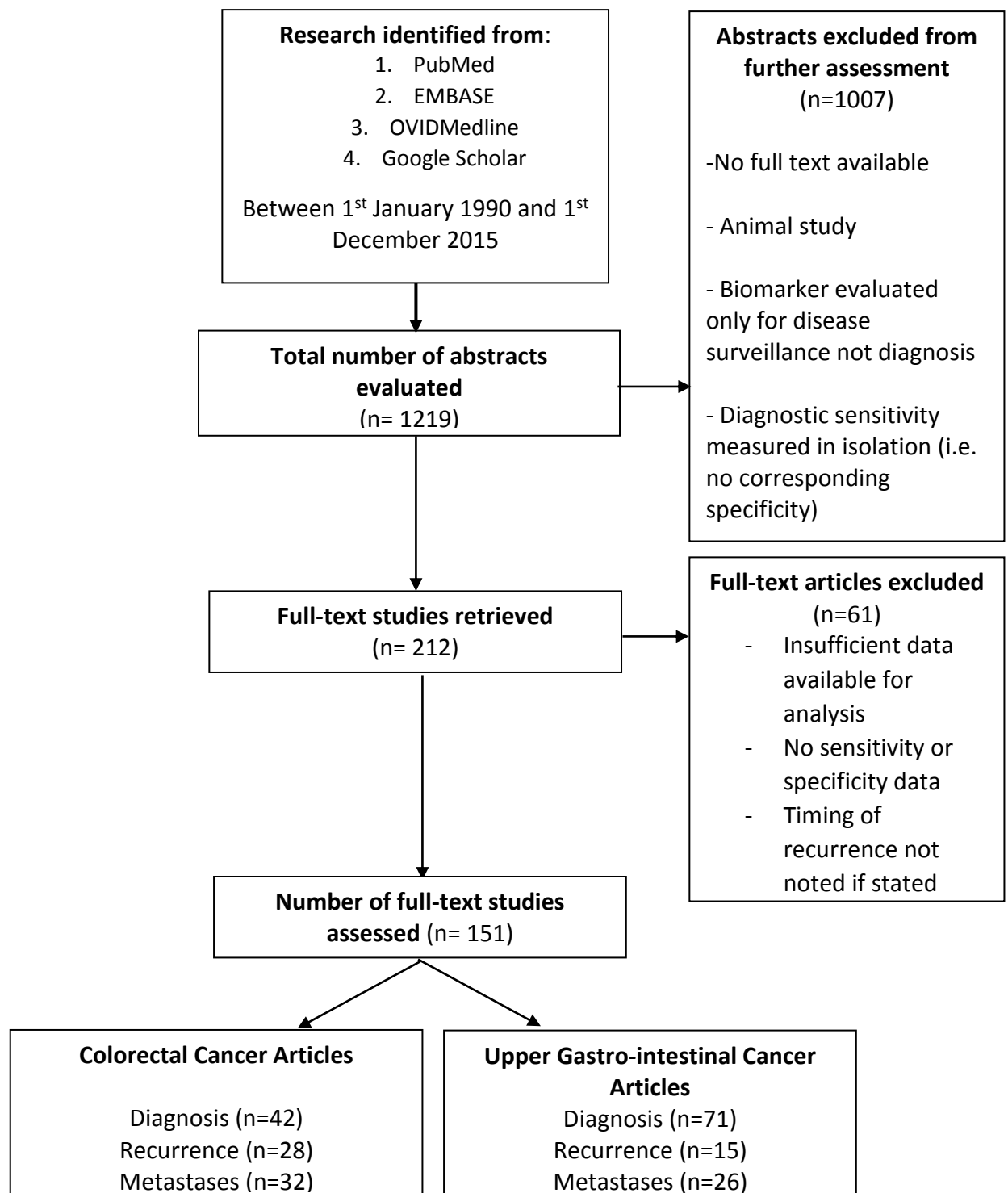

Supplement: Supplementary file 1 — Supplementary material 1 (PDF 181 kb) [file 10434_2016_5717_MOESM1_ESM.pdf]
