# Supplementary material for: Use of Tumor Markers in Gastrointestinal Cancers: Surgeon Perceptions and Cost-Benefit Trade-Off Analysis
Source: Ann Surg Oncol. 2016 Dec 22;24(5):1165–73. doi: 10.1245/s10434-016-5717-y (PMC5374165; doi:10.1245/s10434-016-5717-y)
Supplement: Supplementary file 2 — Supplementary material 2 (PDF 226 kb) [file 10434_2016_5717_MOESM2_ESM.pdf]

Appendix 2: Survey distributed to European surgeons originally anonymised and online, demonstrating survey mechanics.

Q1 What best describes your current level/role?
- ❍ Consultant/Attending/Professor (1)
- ❍ Resident/Registrar (2)
- ❍ Intern (3)
- ❍ Academic/Researcher (4)

Q3 Please select which sub-specialty you primarily work in?
- ❍ Upper Gastro-Intestinal (including esophageal and gastric) (1)
- ❍ Lower Gastro-Intestinal (including colorectal and proctology) (2)
- ❍ Hepato-Biliary (including transplant) (3)

Answer If Please select which sub-specialty you primarily work in? Upper Gastro-Intestinal (including esophageal and gastric) Is Selected Or Do you have any surgical experience of any other sub-specialty? Yes- Upper Gastro-Intestinal (including oesophageal and gastric) Is Selected

Q4 Please state how many cancer operations (esophagectomy or gastrectomy) you have surgical experience (either as primary operator or first assistant) of?
- ❍ Less than 50 operations (1)
- ❍ Between 50 and 100 operations (2)
- ❍ More than 100 operations (3)

Answer If Please select which sub-specialty you primarily work in? Lower Gastro-Intestinal (including colorectal and proctology) Is Selected Or Do you have any surgical experience of any other sub-specialty? Yes- Lower Gastro-Intestinal (including colorectal and proctology) Is Selected

Q5 Please state how many cancer operations (including anterior resections, abdomino-perineal resections and total meso-rectal excisions) you have surgical experience (either as primary operator or first assistant) of?
- ❍ Less than 50 operations (1)
- ❍ Between 50 and 100 operations (2)
- ❍ More than 100 operations (3)

Answer If Please select which sub-specialty you primarily work in? Hepato-Biliary (including transplant) Is Selected

Q6 Do you have any surgical experience of any other sub-specialty?
- ❍ Yes- Upper Gastro-Intestinal (including esophageal and gastric) (1)
- ❍ Yes- Lower Gastro-Intestinal (including colorectal and proctology) (2)
- ❍ No (3)

If No Is Selected, Then Skip To End of Survey

Q7 Which of the of the following tumor markers do you commonly use (for any purpose) in your routine practice? You may select more than one.

- ❑ Carcino-embryonic antigen (CEA) (1)
- ❑ CA 19-9 (2)
- ❑ Ca 125 (3)
- ❑ CD34 (4)
- ❑ Alpha Feto-Protein (AFP) (5)
- ❑ I do not commonly use tumor markers (6)

If I do not commonly use tumou... Is Selected, Then Skip To Please rank the attributes or charact...

Q8 What do you use tumor markers primarily for?

- ❍ Diagnosis of cancer (1)
- ❍ Monitor for recurrence (2)
- ❍ Assess severity (for example presence of metastasis) (3)
- ❍ Determine difficulty of operation (4)
- ❍ Assess prognosis (5)

Q9 Please rank the attributes or characteristics that you would want from the IDEAL tumor marker. DRAG the most desirable to the top and least desirable to the bottom.

______ High Sensitivity (1)
______ High Specificity (2)
______ The ability to predict recurrence (3)
______ The ability to detect metastasis (4)
______ Low cost (5)
______ Quick to analyze result (6)
______ Consistent across demographics (7)
______ Acceptable test for the patient (8)
