# Supplementary material for: Use of Tumor Markers in Gastrointestinal Cancers: Surgeon Perceptions and Cost-Benefit Trade-Off Analysis
Source: Ann Surg Oncol. 2016 Dec 22;24(5):1165–73. doi: 10.1245/s10434-016-5717-y (PMC5374165; doi:10.1245/s10434-016-5717-y)
Supplement: Supplementary file 3 — Supplementary material 3 (PDF 387 kb) [file 10434_2016_5717_MOESM3_ESM.pdf]

Appendix 3: Full references for all included studies.

1. Aggarwal C, Meropol NJ, Punt CJ et al. Relationship among circulating tumor cells, CEA and overall survival in patients with metastatic colorectal cancer. *Annals of Oncology : Official Journal of the European Society for Medical Oncology / ESMO* 2013 Feb;24(2): pp. 420-428.

2. Alici S, Ugras S, Bayram I, Izmirli M. Prognostic factors and COX-2 expression in advanced stage esophageal squamous cell carcinoma. *Advances in Therapy* 2006;23(5): pp. 672-679.

3. Alvarez J, Marín J, Jover JM, Fernandez R, Fradejas J, Moreno M. Sensitivity of monoclonal antibodies to carcinoembryonic antigen, tissue polypeptide antigen, alpha-fetoprotein, carbohydrate antigen 50, and carbohydrate antigen 19-9 in the diagnosis of colorectaladenocarcinoma. *Diseases of the Colon and the Rectum* 1995;38(5): pp. 535-542.

4. Bagaria B, Sood S, Sharma R, Lalwani S. Comparative study of CEA and CA19-9 in esophageal, gastric and colon cancers individually and in combination (ROC curve analysis). *Cancer biology & medicine* 2013 Sep;10(3): pp. 148-157.

5. Bakalakos EA, Burak WE,Jr, Young DC, Martin EW. Is carcino-embryonic antigen useful in the follow-up management of patients with colorectal liver metastases? *American Journal of Surgery* 1999 Jan;177(1): pp. 2-6.

6. Banki F, Yacoub WN, Hagen JA et al. Plasma DNA is more reliable than carcinoembryonic antigen for diagnosis of recurrent esophageal cancer. *Journal of the American College of Surgeons* 2008 Jul;207(1): pp. 30-35.

7. Bao J, Qu G, Fu W et al. Clinicopathological features of gastric adenocarcinoma patients with metachronous distant metastasis. *Tumour biology : the journal of the International Society for Oncodevelopmental Biology and Medicine* 2015 Aug;36(8): pp. 6375-6382.

8. Barillari P, Sammartino P, Cardi M et al. Gastrointestinal cancer follow-up: the effectiveness of sequential CEA, TPA and Ca 19-9 evaluation in the early diagnosis of recurrences. *The Australian and New Zealand Journal of Surgery* 1991 Sep;61(9): pp. 675-680.

9. Bold RJ, Ota DM, Ajani JA, Mansfield PF. Peritoneal and serum tumor markers predict recurrence and survival of patients with resectable gastric cancer. *Gastric cancer : official journal of the International Gastric Cancer Association and the Japanese Gastric Cancer Association* 1999 May;2(1): pp. 1-7.

10. Bonfanti G, Bombelli L, Bozzetti F, Doci R, Gennari L, Koukouras D. The role of CEA and liver function tests in the detection of hepatic metastases from colo-rectal cancer. *HPB surgery : a world journal of hepatic, pancreatic and biliary surgery* 1990;3(1): pp. 29-36; discussion 36-7.

11. Bu W, Wei R, Li J et al. Association between carcinoembryonic antigen levels and the applied value of F-fluorodeoxyglucose positron emission tomography/computed tomography in post-operative recurrent and metastatic colorectal cancer. *Oncology letters* 2014 Dec;8(6): pp. 2649-2653.

12. Caglar M, Yener C, Karabulut E. Value of CT, FDG PET-CT and serum tumor markers in staging recurrent colorectal cancer. *International journal of computer assisted radiology and surgery* 2015 Jul;10(7): pp. 993-1002.

13. Carpelan-Holmstrom M, Haglund C, Lundin J, Alfthan H, Stenman UH, Roberts PJ . Independent prognostic value of preoperative serum markers CA 242, specific tissue polypeptide antigen and human chorionic gonadotrophin beta, but not of carcinoembryonic antigen or tissue polypeptide antigen in colorectal cancer. *British journal of cancer* 1996 Sep;74(6): pp. 925-929.

14. Carpelan-Holmstrom M, Louhimo J, Stenman UH, Alfthan H, Haglund C. CEA, CA 19-9 and CA 72-4 improve the diagnostic accuracy in gastrointestinal cancers. *Anticancer Research* 2002 Jul-Aug;22(4): pp. 2311-2316.

15. Castaldi F, Marino M, Beneduce L et al. Detection of circulating CEA-IgM complexes in early stage colorectal cancer. *The International journal of biological markers* 2005 Oct-Dec;20(4): pp. 204-208.

16. Cetin B, Atalay C, Aslan S et al. Peritoneal carcinoembryonic antigen level for predicting locoregional and distant spread of gastric cancer. *Surgery today* 2005;35(11): pp. 919-924.

17. Chan AO, Chu KM, Lam SK et al. Early prediction of tumor recurrence after curative resection of gastric carcinoma by measuring soluble E-cadherin. *Cancer* 2005 Aug 15;104(4): pp. 740-746.

18. Chan CC, Fan CW, Kuo YB et al. Multiple serological biomarkers for colorectal cancer detection. *International journal of cancer.Journal international du cancer* 2010 Apr 1;126(7): pp. 1683-1690.

19. Chen C, Chen LQ, Yang G, Li Y. Value of tumor markers in diagnosing and monitoring colorectal cancer and strategies for further improvement: analysis of 130 cases. *Ai zheng = Aizheng = Chinese journal of cancer* 2007 Nov;26(11): pp. 1221-1226.

20. Chen S, Feng XY, Li YF, Zhao BW, Zhou ZW, Chen YB. The prognosis of gastric cancer patients with marginally elevated carcinoembryonic antigen (CEA) values after D2 radical gastrectomy. *Journal of surgical oncology* 2013 May;107(6): pp. 641-645.

21. Chester SJ, Maimonis P, Vanzuiden P, Finkelstein M, Bookout J, Vezeridis MP. A new radioimmunoassay detecting early stages of colon cancer: a comparison with CEA, AFP, and Ca 19-9. *Disease markers* 1991 Sep-Oct;9(5): pp. 265-271.

22. Cho S, Song IH, Yang HC, Jheon S. Prognostic factors of pulmonary metastasis from colorectal carcinoma. *Interactive cardiovascular and thoracic surgery* 2013 Aug;17(2): pp. 303-307.

23. Cidon EU, Bustamante R. Gastric cancer: tumor markers as predictive factors for preoperative staging. *Journal of gastrointestinal cancer* 2011 Sep;42(3): pp. 127-130.

24. Clark GW, Ireland AP, Hagen JA, Collard JM, Peters JH, DeMeester TR. Carcinoembryonic antigen measurements in the management of esophageal cancer: an indicator of subclinical recurrence. *American Journal of Surgery* 1995 Dec;170(6): pp. 597-600; discussion 600-1.

25. Dong H, Tang J, Li LH et al. Serum carbohydrate antigen 19-9 as an indicator of liver metastasis in colorectal carcinoma cases. *Asian Pacific journal of cancer prevention : APJCP* 2013;14(2): pp. 909-913.

26. Duraker N, Celik AN. The prognostic significance of preoperative serum CA 19-9 in patients with resectable gastric carcinoma: comparison with CEA. *Journal of surgical oncology* 2001 Apr;76(4): pp. 266-271.

27. Eker B, Ozaslan E, Karaca H et al. Factors affecting prognosis in metastatic colorectal cancer patients. *Asian Pacific journal of cancer prevention : APJCP* 2015;16(7): pp. 3015-3021.

28. Eleftheriadis N, Papaloukas C, Pistevou-Gompaki K. Diagnostic value of serum tumor markers in asymptomatic individuals. *Journal of B.U.ON.: official journal of the Balkan Union of Oncology* 2009 Oct-Dec;14(4): pp. 707-710.

29. Emoto S, Ishigami H, Yamashita H, Yamaguchi H, Kaisaki S, Kitayama J. Clinical significance of CA125 and CA72-4 in gastric cancer with peritoneal dissemination. *Gastric cancer : official journal of the International Gastric Cancer Association and the Japanese Gastric Cancer Association* 2012 Apr;15(2): pp. 154-161.

30. Engaras B. Individual cutoff levels of carcinoembryonic antigen and CA 242 indicate recurrence of colorectal cancer with high sensitivity. *Diseases of the colon and rectum* 2003 Mar;46(3): pp. 313-321.

31. Fiala O, Finek J, Buchler T et al. The Association of Serum Carcinoembryonic Antigen, Carbohydrate Antigen 19-9, Thymidine Kinase, and Tissue Polypeptide Specific Antigen with Outcomes of Patients with Metastatic Colorectal Cancer Treated with Bevacizumab: a Retrospective Study. *Targeted oncology* 2015 Dec;10(4): pp. 549-555.

32. Fujimura T, Kinami S, Ninomiya I et al. Diagnostic laparoscopy, serum CA125, and peritoneal metastasis in gastric cancer. *Endoscopy* 2002 Jul;34(7): pp. 569-574.

33. Giessen C, Nagel D, Glas M et al. Evaluation of preoperative serum markers for individual patient prognosis in stage I-III rectal cancer. *Tumour biology : the journal of the International Society for Oncodevelopmental Biology and Medicine* 2014 Oct;35(10): pp. 10237-10248.

34. Giessen-Jung C, Nagel D, Glas M et al. Preoperative serum markers for individual patient prognosis in stage I-III colon cancer. *Tumour biology : the journal of the International Society for Oncodevelopmental Biology and Medicine* 2015 Sep;36(10): pp. 7897-7906.

35. Glover C, Douse P, Kane P et al. Accuracy of investigations for asymptomatic colorectal liver metastases. *Diseases of the colon and rectum* 2002 Apr;45(4): pp. 476-484.

36. Gwak HK, Lee JH, Park SG. Preliminary evaluation of clinical utility of CYFRA 21-1, CA 72-4, NSE, CA19-9 and CEA in stomach cancer. *Asian Pacific journal of cancer prevention : APJCP* 2014;15(12): pp. 4933-4938.

37. Haglund C, Ylatupa S, Mertaniemi P, Partanen P. Cellular fibronectin concentration in the plasma of patients with malignant and benign diseases: a comparison with CA 19-9 and CEA. *British journal of cancer* 1997;76(6): pp. 777-783.

38. Hakama M, Stenman UH, Knekt P et al. Tumour markers and screening for gastrointestinal cancer: a follow up study in Finland. *Journal of medical screening* 1994 Jan;1(1): pp. 60-64.

39. Hall NR, Finan PJ, Stephenson BM, Purves DA, Cooper EH. The role of CA-242 and CEA in surveillance following curative resection for colorectal cancer. *British journal of cancer* 1994 Sep;70(3): pp. 549-553.

40. Hao TB, Shi W, Shen XJ et al. Circulating cell-free DNA in serum as a biomarker for diagnosis and prognostic prediction of colorectal cancer. *British journal of cancer* 2014 Oct 14;111(8): pp. 1482-1489.

41. He CZ, Zhang KH, Li Q, Liu XH, Hong Y, Lv NH. Combined use of AFP, CEA, CA125 and CAl9-9 improves the sensitivity for the diagnosis of gastric cancer. *BMC gastroenterology* 2013 May 14;13: pp. 87-230X-13-87.

42. Huang SC, Lin JK, Lin TC et al. Concordance of Carcinoembryonic Antigen Ratio and Response Evaluation Criteria in Solid Tumors as Prognostic Surrogate Indicators of Metastatic Colorectal Cancer Patients Treated with Chemotherapy. *Annals of surgical oncology* 2015 Jul;22(7): pp. 2262-2268.

43. Huang ZB, Zhou X, Xu J et al. Prognostic value of preoperative serum tumor markers in gastric cancer. *World journal of clinical oncology* 2014 May 10;5(2): pp. 170-176.

44. Ikeda M, Natsugoe S, Ueno S, Baba M, Aikou T. Significant host- and tumor-related factors for predicting prognosis in patients with esophageal carcinoma. *Annals of Surgery* 2003 Aug;238(2): pp. 197-202.

45. Inoue H, Kajiyama Y, Tsurumaru M. Clinical significance of bone marrow micrometastases in esophageal cancer. *Diseases of the esophagus : official journal of the International Society for Diseases of the Esophagus / I.S.D.E* 2004;17(4): pp. 328-332.

46. Irvine T, Scott M, Clark CI. A small rise in CEA is sensitive for recurrence after surgery for colorectal cancer. *Colorectal disease : the official journal of the Association of Coloproctology of Great Britain and Ireland* 2007 Jul;9(6): pp. 527-531.

47. Ishigami S, Natsugoe S, Hokita S et al. Clinical importance of preoperative carcinoembryonic antigen and carbohydrate antigen 19-9 levels in gastric cancer. *Journal of clinical gastroenterology* 2001 Jan;32(1): pp. 41-44.

48. Ishizuka D, Shirai Y, Sakai Y, Hatakeyama K. Colorectal carcinoma liver metastases: clinical significance of preoperative measurement of serum carcinoembryonic antigen and carbohydrate antigen 19-9 levels. *International journal of colorectal disease* 2001 Feb;16(1): pp. 32-37.

49. Jain P, Mondal SK, Sinha SK, Mukhopadhyay M, Chakraborty I. Diagnostic and prognostic significance of different mucin expression, preoperative CEA, and CA-125 in colorectal carcinoma: A clinicopathological study. *Journal of natural science, biology, and medicine* 2014 Jul;5(2): pp. 404-408.

50. Jeong JY, Kim MG, Ha TK, Kwon SJ. Prognostic factors on overall survival in lymph node negative gastric cancer patients who underwent curative resection. *Journal of gastric cancer* 2012 Dec;12(4): pp. 210-216.

51. Jing JX, Wang Y, Xu XQ et al. Tumor markers for diagnosis, monitoring of recurrence and prognosis in patients with upper gastrointestinal tract cancer. *Asian Pacific journal of cancer prevention : APJCP* 2014;15(23): pp. 10267-10272.

52. Jo JC, Ryu MH, Koo DH et al. Serum CA 19-9 as a prognostic factor in patients with metastatic gastric cancer. *Asia-Pacific journal of clinical oncology* 2013 Dec;9(4): pp. 324-330.

53. Jones C, Badger SA, Epanomeratikis E, McKie LD, Diamond T, Taylor MA. Role of carcinoembryonic antigen as a marker for colorectal liver metastases. *British journal of biomedical science* 2013;70(2): pp. 47-50.

54. Kaifi JT, Kunkel M, Dicker DT et al. Circulating tumor cell levels are elevated in colorectal cancer patients with high tumor burden in the liver. *Cancer biology & therapy* 2015;16(5): pp. 690-698.

55. Kanellos I, Zacharakis E, Demetriades H et al. Value of carcinoembryonic antigen assay in predicting hepatic metastases, local recurrence, and survival after curative resection of colorectal cancer. *Surgery today* 2006;36(10): pp. 879-884.

56. Kawamura YJ, Tokumitsu A, Sasaki J et al. Colorectal carcinoma with extremely low CA19-9. *Gastroenterology research and practice* 2009;2009: pp. 780263.

57. Khanfir A, Feki J, Zidi Z et al. Prognostic factors in metastatic colorectal cancer in Tunisia: A retrospective study of 130 patients. *La Tunisie medicale* 2015 Jan;93(1): pp. 11-15.

58. Kijima M, Togo S, Ichikawa Y et al. Clinical significance of serum CEA protein and CEA mRNA after resection of colorectal liver metastases. *Anticancer Research* 2005 Mar-Apr;25(2B): pp. 1327-1332.

59. Kim DH, Yun HY, Ryu DH et al. Preoperative CA 125 is significant indicator of curative resection in gastric cancer patients. *World journal of gastroenterology* 2015 Jan 28;21(4): pp. 1216-1221.

60. Kim JP, Lee JH, Kim SJ, Yu HJ, Yang HK. Clinicopathologic characteristics and prognostic factors in 10 783 patients with gastric cancer. *Gastric cancer : official journal of the International Gastric Cancer Association and the Japanese Gastric Cancer Association* 1998 Mar;1(2): pp. 125-133.

61. Kim YH, Ajani JA, Ota DM, Lynch P, Roth JA. Value of serial carcinoembryonic antigen levels in patients with resectable adenocarcinoma of the esophagus and stomach. *Cancer* 1995 Jan 15;75(2): pp. 451-456.

62. Kochi M, Fujii M, Kanamori N et al. Evaluation of serum CEA and CA19-9 levels as prognostic factors in patients with gastric cancer. *Gastric cancer : official journal of the International Gastric Cancer Association and the Japanese Gastric Cancer Association* 2000 Dec;3(4): pp. 177-186.

63. Kornek GV, Depisch D, Rosen HR, Temsch EM, Scheithauer W. Comparative analysis of CA72-4, CA195 and carcinoembryonic antigen in patients with gastrointestinal malignancies. *Journal of cancer research and clinical oncology* 1992;118(4): pp. 318-320.

64. Korner H, Soreide K, Stokkeland PJ, Soreide JA. Systematic follow-up after curative surgery for colorectal cancer in Norway: a population-based audit of effectiveness, costs, and compliance. *Journal of gastrointestinal surgery : official journal of the Society for Surgery of the Alimentary Tract* 2005 Mar;9(3): pp. 320-328.

65. Kosmider S, Stella DL, Field K et al. Preoperative investigations for metastatic staging of colon and rectal cancer across multiple centres--what is current practice? *Colorectal disease : the official journal of the Association of Coloproctology of Great Britain and Ireland* 2009 Jul;11(6): pp. 592-600.

66. Kosugi S, Nishimaki T, Kanda T, Nakagawa S, Ohashi M, Hatakeyama K. Clinical significance of serum carcinoembryonic antigen, carbohydrate antigen 19-9, and squamous cell carcinoma antigen levels in esophageal cancer patients. *World journal of surgery* 2004 Jul;28(7): pp. 680-685.

67. Kuusela P, Haglund C, Roberts PJ. Comparison of a new tumour marker CA 242 with CA 19-9, CA 50 and carcinoembryonic antigen (CEA) in digestive tract diseases. *British journal of cancer* 1991 Apr;63(4): pp. 636-640.

68. Lai H, Jin Q, Lin Y et al. Combined use of lysyl oxidase, carcino-embryonic antigen, and carbohydrate antigens improves the sensitivity of biomarkers in predicting lymph node metastasis and peritoneal metastasis in gastric cancer. *Tumour biology : the journal of the International Society for Oncodevelopmental Biology and Medicine* 2014 Oct;35(10): pp. 10547-10554.

69. Lee DY, Hong SW, Chang YG, Lee WY, Lee B. Clinical significance of preoperative inflammatory parameters in gastric cancer patients. *Journal of gastric cancer* 2013 Jun;13(2): pp. 111-116.

70. Lee EC, Yang JY, Lee KG et al. The value of postoperative serum carcinoembryonic antigen and carbohydrate antigen 19-9 levels for the early detection of gastric cancer recurrence after curative resection. *Journal of gastric cancer* 2014 Dec;14(4): pp. 221-228.

71. Lee JC, Lee SY, Kim CY, Yang DH. Clinical utility of tumor marker cutoff ratio and a combination scoring system of preoperative carcinoembryonic antigen, carbohydrate

antigen 19-9, carbohydrate antigen 72-4 levels in gastric cancer. *Journal of the Korean Surgical Society* 2013 Dec;85(6): pp. 283-289.

72. Li F, Li S, Wei L, Liang X, Zhang H, Liu J. The correlation between pre-operative serum tumor markers and lymph node metastasis in gastric cancer patients undergoing curative treatment. *Biomarkers : biochemical indicators of exposure, response, and susceptibility to chemicals* 2013 Nov;18(7): pp. 632-637.

73. Li Y, Yang Y, Lu M, Shen L. Predictive value of serum CEA, CA19-9 and CA72.4 in early diagnosis of recurrence after radical resection of gastric cancer. *Hepato-gastroenterology* 2011 Nov-Dec;58(112): pp. 2166-2170.

74. Lim JB, Chung HW. Serum ENA78/CXCL5, SDF-1/CXCL12, and their combinations as potential biomarkers for prediction of the presence and distant metastasis of primary gastric cancer. *Cytokine* 2015 May;73(1): pp. 16-22.

75. Lindmark G, Bergstrom R, Pahlman L, Glimelius B. The association of preoperative serum tumour markers with Dukes' stage and survival in colorectal cancer. *British journal of cancer* 1995 May;71(5): pp. 1090-1094.

76. Liu J, Huang XE. Clinical application of serum tumor abnormal protein from patients with gastric cancer. *Asian Pacific journal of cancer prevention : APJCP* 2015;16(9): pp. 4041-4044.

77. Liu X, Cai H, Wang Y. Prognostic significance of tumour markers in Chinese patients with gastric cancer. *ANZ Journal of Surgery* 2014 Jun;84(6): pp. 448-453.

78. Lopez JB, Royan GP, Lakhwani MN, Mahadevan M, Timor J. CA 72-4 compared with CEA and CA 19-9 as a marker of some gastrointestinal malignancies. *The International journal of biological markers* 1999 Jul-Sep;14(3): pp. 172-177.

79. Lukaszewicz-Zajac M, Mroczko B, Kozlowski M et al. Comparative evaluation of serum C-reactive protein (CRP) levels in the different histological subtypes of esophageal cancer (squamous cell carcinoma and adenocarcinoma of esophagus). *Journal of clinical laboratory analysis* 2012 Feb;26(2): pp. 73-81.

80. Maehara Y, Sugimachi K, Akagi M, Kakegawa T, Shimazu H, Tomita M. Serum carcinoembryonic antigen level increases correlate with tumor progression in patients with differentiated gastric carcinoma following noncurative resection. *Cancer research* 1990 Jul 1;50(13): pp. 3952-3955.

81. Marrelli D, Pinto E, De Stefano A et al. Preoperative positivity of serum tumor markers is a strong predictor of hematogenous recurrence of gastric cancer. *Journal of surgical oncology* 2001 Dec;78(4): pp. 253-258.

82. Martell RE, Xu FJ, Davis WZ et al. OVX1 and CEA in patients with colon carcinoma, colon polyps and benign colon disorders. *The International journal of biological markers* 1998 Jul-Sep;13(3): pp. 145-149.

83. Mattar R, Alves de Andrade CR, DiFavero GM, Gama-Rodrigues JJ, Laudanna AA. Preoperative serum levels of CA 72-4, CEA, CA 19-9, and alpha-fetoprotein in patients with gastric cancer. *Revista do Hospital das Clinicas* 2002 May-Jun;57(3): pp. 89-92.

84. McCall JL, Black RB, Rich CA et al. The value of serum carcinoembryonic antigen in predicting recurrent disease following curative resection of colorectal cancer. *Diseases of the colon and rectum* 1994 Sep;37(9): pp. 875-881.

85. McKnight A, Mannell A, Shperling I. The role of carbohydrate antigen 19-9 as a tumour marker of oesophageal cancer. *British journal of cancer* 1989 Aug;60(2): pp. 249-251.

86. Migita K, Takayama T, Saeki K et al. The prognostic nutritional index predicts long-term outcomes of gastric cancer patients independent of tumor stage. *Annals of surgical oncology* 2013 Aug;20(8): pp. 2647-2654.

87. Moertel CG, Fleming TR, Macdonald JS, Haller DG, Laurie JA, Tangen C. An evaluation of the carcinoembryonic antigen (CEA) test for monitoring patients with resected colon cancer. *Jama* 1993 Aug 25;270(8): pp. 943-947.

88. Morita S, Nomura T, Fukushima Y, Morimoto T, Hiraoka N, Shibata N. Does serum CA19-9 play a practical role in the management of patients with colorectal cancer? *Diseases of the colon and rectum* 2004 Feb;47(2): pp. 227-232.

89. Motavaf E, Sunesen KG, Stender MT, Thorlacius-Ussing O. Prognostic value of preoperative D-dimer and carcinoembryonic antigen levels in patients undergoing intended curative resection for colorectal cancer: a prospective cohort study. *International journal of colorectal disease* 2014 Nov;29(11): pp. 1427-1432.

90. Mourtzikou A, Stamouli M, Kroupis C et al. Evaluation of carcinoembryonic antigen (CEA), epidermal growth factor receptor (EGFR), epithelial cell adhesion molecule EpCAM (GA733-2), and carbohydrate antigen 19-9 (CA 19-9) levels in colorectal cancer

patients and correlation with clinicopathological characteristics. *Clinical laboratory* 2012;58(5-6): pp. 441-448.

91. Mroczko B, Kozlowski M, Groblewska M et al. The diagnostic value of the measurement of matrix metalloproteinase 9 (MMP-9), squamous cell cancer antigen (SCC) and carcinoembryonic antigen (CEA) in the sera of esophageal cancer patients. *Clinica chimica acta; international journal of clinical chemistry* 2008 Mar;389(1-2): pp. 61-66.

92. Nakashima S, Natsugoe S, Matsumoto M et al. Biological properties of biopsy specimens are useful for predicting lymph node micrometastasis in esophageal carcinoma. *Anticancer Research* 2002 Sep-Oct;22(5): pp. 2951-2956.

93. Nakayama T, Watanabe M, Teramoto T, Kitajima M. CA19-9 as a predictor of recurrence in patients with colorectal cancer. *Journal of surgical oncology* 1997 Dec;66(4): pp. 238-243.

94. Narita M, Oussoultzoglou E, Chenard MP et al. Predicting early intrahepatic recurrence after curative resection of colorectal liver metastases with molecular markers. *World journal of surgery* 2015 May;39(5): pp. 1167-1176.

95. Ohno S, Fujii T, Ueda S et al. Predictive factors and timing for liver recurrence after curative resection of gastric carcinoma. *American Journal of Surgery* 2003 Mar;185(3): pp. 258-263.

96. Ohtsuka T, Nakafusa Y, Sato S, Kitajima Y, Tanaka M, Miyazaki K. Different roles of tumor marker monitoring after curative resections of gastric and colorectal cancers. *Digestive diseases and sciences* 2008 Jun;53(6): pp. 1537-1543.

97. Oshima Y, Shimada H, Yajima S et al. NY-ESO-1 autoantibody as a tumor-specific biomarker for esophageal cancer: screening in 1969 patients with various cancers. *Journal of gastroenterology* 2015 Apr 24.

98. Oussoultzoglou E, Rosso E, Fuchshuber P et al. Perioperative carcinoembryonic antigen measurements to predict curability after liver resection for colorectal metastases: a prospective study. *Archives of surgery (Chicago, Ill.: 1960)* 2008 Dec;143(12): pp. 1150-8; discussion 1158-9.

99. Park IJ, Choi GS, Jun SH. Prognostic value of serum tumor antigen CA19-9 after curative resection of colorectal cancer. *Anticancer Research* 2009 Oct;29(10): pp. 4303-4308.

100. Park IJ, Choi GS, Lim KH, Kang BM, Jun SH. Serum carcinoembryonic antigen monitoring after curative resection for colorectal cancer: clinical significance of the preoperative level. *Annals of surgical oncology* 2009 Nov;16(11): pp. 3087-3093.

101. Patt YZ, Lamki LM, Shanken J et al. Imaging with indium111-labeled anticarcinoembryonic antigen monoclonal antibody ZCE-025 of recurrent colorectal or carcinoembryonic antigen-producing cancer in patients with rising serum carcinoembryonic antigen levels and occult metastases. *Journal of clinical oncology : official journal of the American Society of Clinical Oncology* 1990 Jul;8(7): pp. 1246-1254.

102. Pectasides D, Mylonakis A, Kostopoulou M et al. CEA, CA 19-9, and CA-50 in monitoring gastric carcinoma. *American journal of clinical oncology* 1997 Aug;20(4): pp. 348-353.

103. Peng Y, Zhai Z, Li Z, Wang L, Gu J. Role of blood tumor markers in predicting metastasis and local recurrence after curative resection of colon cancer. *International journal of clinical and experimental medicine* 2015 Jan 15;8(1): pp. 982-990.

104. Polat E, Duman U, Duman M et al. Diagnostic value of preoperative serum carcinoembryonic antigen and carbohydrate antigen 19-9 in colorectal cancer. *Current oncology (Toronto, Ont.)* 2014 Feb;21(1): pp. e1-7.

105. Sato H, Usuda N, Kuroda M, Hashimoto S, Maruta M, Maeda K. Significance of serum concentrations of E-selectin and CA19-9 in the prognosis of colorectal cancer. *Japanese journal of clinical oncology* 2010 Nov;40(11): pp. 1073-1080.

106. Scarpa M, Noaro G, Saadeh L et al. Esophageal cancer management: preoperative CA19.9 and CEA serum levels may identify occult advanced adenocarcinoma. *World journal of surgery* 2015 Feb;39(2): pp. 424-432.

107. Schneebaum S, Arnold MW, Young D et al. Role of carcinoembryonic antigen in predicting resectability of recurrent colorectal cancer. *Diseases of the colon and rectum* 1993 Sep;36(9): pp. 810-815.

108. Schneider J, Bitterlich N, Schulze G. Improved sensitivity in the diagnosis of gastro-intestinal tumors by fuzzy logic-based tumor marker profiles including the tumor M2-PK. *Anticancer Research* 2005 May-Jun;25(3A): pp. 1507-1515.

109. Seifert JK, Morris DL. Prognostic factors after cryotherapy for hepatic metastases from colorectal cancer. *Annals of Surgery* 1998 Aug;228(2): pp. 201-208.

110. Selcukbiricik F, Bilici A, Tural D et al. Are high initial CEA and CA 19-9 levels associated with the presence of K-ras mutation in patients with metastatic colorectal cancer? *Tumour biology : the journal of the International Society for Oncodevelopmental Biology and Medicine* 2013 Aug;34(4): pp. 2233-2239.

111. Setoyama T, Natsugoe S, Okumura H et al. Carcinoembryonic antigen messenger RNA expression in blood predicts recurrence in esophageal cancer. *Clinical cancer research : an official journal of the American Association for Cancer Research* 2006 Oct 15;12(20 Pt 1): pp. 5972-5977.

112. Shimada H, Nabeya Y, Okazumi S et al. Prediction of survival with squamous cell carcinoma antigen in patients with resectable esophageal squamous cell carcinoma. *Surgery* 2003 May;133(5): pp. 486-494.

113. Shimada H, Takeda A, Arima M et al. Serum p53 antibody is a useful tumor marker in superficial esophageal squamous cell carcinoma. *Cancer* 2000 Oct 15;89(8): pp. 1677-1683.

114. Shu J, Li CG, Liu YC et al. Comparison of serum tumor associated material (TAM) with conventional biomarkers in cancer patients. *Asian Pacific journal of cancer prevention : APJCP* 2012;13(5): pp. 2399-2403.

115. Sisik A, Kaya M, Bas G, Basak F, Alimoglu O. CEA and CA 19-9 are still valuable markers for the prognosis of colorectal and gastric cancer patients. *Asian Pacific journal of cancer prevention : APJCP* 2013;14(7): pp. 4289-4294.

116. Solakidi S, Dessypris A, Stathopoulos GP, Androulakis G, Sekeris CE. Tumour-associated trypsin inhibitor, carcinoembryonic antigen and acute-phase reactant

proteins CRP and alpha1-antitrypsin in patients with gastrointestinal malignancies. *Clinical biochemistry* 2004 Jan;37(1): pp. 56-60.

117. Spindler KL, Pallisgaard N, Andersen RF, Brandslund I, Jakobsem A. Circulating free DNA as biomarker and source for mutation detection in metastatic colorectal cancer. *PloS one* 2015 Apr 13;10(4): pp. e0108247.

118. Stiksma J, Grootendorst DC, van der Linden PW. CA 19-9 as a marker in addition to CEA to monitor colorectal cancer. *Clinical colorectal cancer* 2014 Dec;13(4): pp. 239-244.

119. Streppel MM, Vincent A, Mukherjee R et al. Mucin 16 (cancer antigen 125) expression in human tissues and cell lines and correlation with clinical outcome in adenocarcinomas of the pancreas, esophagus, stomach, and colon. *Human pathology* 2012 Oct;43(10): pp. 1755-1763.

120. Sun ZQ, Han XN, Wang HJ et al. Prognostic significance of preoperative fibrinogen in patients with colon cancer. *World journal of gastroenterology* 2014 Jul 14;20(26): pp. 8583-8591.

121. Tachibana M, Takemoto Y, Nakashima Y et al. Serum carcinoembryonic antigen as a prognostic factor in resectable gastric cancer. *Journal of the American College of Surgeons* 1998 Jul;187(1): pp. 64-68.

122. Takagawa R, Fujii S, Ohta M et al. Preoperative serum carcinoembryonic antigen level as a predictive factor of recurrence after curative resection of colorectal cancer. *Annals of surgical oncology* 2008 Dec;15(12): pp. 3433-3439.

123. Takahashi K, Miyashita M, Nomura T et al. Serum p53 antibody as a predictor of early recurrence in patients with postoperative esophageal squamous cell carcinoma. *Diseases of the esophagus : official journal of the International Society for Diseases of the Esophagus / I.S.D.E* 2007;20(2): pp. 117-122.

124. Takakura Y, Ikeda S, Imaoka Y, Urushihara T, Itamoto T. An elevated preoperative serum carbohydrate antigen 19-9 level is a significant predictor for peritoneal dissemination and poor survival in colorectal cancer. *Colorectal disease : the official journal of the Association of Coloproctology of Great Britain and Ireland* 2015 May;17(5): pp. 417-425.

125. Tas F, Faruk Aykan N, Aydiner A, Yasasever V, Topuz E. Measurement of serum CA 19-9 may be more valuable than CEA in prediction of recurrence in patients with gastric cancer. *American journal of clinical oncology* 2001 Apr;24(2): pp. 148-149.

126. Tatli AM, Urakci Z, Kalender ME, Arslan H, Tastekin D, Kaplan MA. Alpha-fetoprotein (AFP) elevation gastric adenocarcinoma and importance of AFP change in tumor response evaluation. *Asian Pacific journal of cancer prevention : APJCP* 2015;16(5): pp. 2003-2007.

127. Tian SB, Yu JC, Kang WM et al. Combined detection of CEA, CA 19-9, CA 242 and CA 50 in the diagnosis and prognosis of resectable gastric cancer. *Asian Pacific journal of cancer prevention : APJCP* 2014;15(15): pp. 6295-6300.

128. Toiyama Y, Inoue Y, Saigusa S et al. C-reactive protein as predictor of recurrence in patients with rectal cancer undergoing chemoradiotherapy followed by surgery. *Anticancer Research* 2013 Nov;33(11): pp. 5065-5074.

129. Tsavaris N, Vonorta K, Tsoutsos H et al. Carcinoembryonic antigen (CEA), alpha-fetoprotein, CA 19.9 and CA 125 in advanced colorectal cancer (ACC). *The International journal of biological markers* 1993 Apr-Jun;8(2): pp. 88-93.

130. Ucar E, Semerci E, Ustun H, Yetim T, Huzmeli C, Gullu M. Prognostic value of preoperative CEA, CA 19-9, CA 72-4, and AFP levels in gastric cancer. *Advances in Therapy* 2008 Oct;25(10): pp. 1075-1084.

131. Ueno H, Konishi T, Ishikawa Y et al. Prognostic value of poorly differentiated clusters in the primary tumor in patients undergoing hepatectomy for colorectal liver metastasis. *Surgery* 2015 May;157(5): pp. 899-908.

132. Verberne CJ, Wiggers T, Vermeulen KM, de Jong KP. Detection of recurrences during follow-up after liver surgery for colorectal metastases: both carcinoembryonic antigen (CEA) and imaging are important. *Annals of surgical oncology* 2013 Feb;20(2): pp. 457-463.

133. Victorzon M, Haglund C, Lundin J, Roberts PJ. A prognostic value of CA 19-9 but not of CEA in patients with gastric cancer. *European journal of surgical oncology : the journal of the European Society of Surgical Oncology and the British Association of Surgical Oncology* 1995 Aug;21(4): pp. 379-384.

134. Wang WS, Lin JK, Chiou TJ et al. Preoperative carcinoembryonic antigen level as an independent prognostic factor in colorectal cancer: Taiwan experience. *Japanese journal of clinical oncology* 2000 Jan;30(1): pp. 12-16.

135. Wang YR, Yan JX, Wang LN. The diagnostic value of serum carcino-embryonic antigen, alpha fetoprotein and carbohydrate antigen 19-9 for colorectal cancer. *Journal of cancer research and therapeutics* 2014 Dec;10 Suppl: pp. 307-309.

136. Webb A, Scott-Mackie P, Cunningham D et al. The prognostic value of CEA, beta HCG, AFP, CA125, CA19-9 and C-erb B-2, beta HCG immunohistochemistry in advanced colorectal cancer. *Annals of Oncology : Official Journal of the European Society for Medical Oncology / ESMO* 1995 Jul;6(6): pp. 581-587.

137. Wiratkapun S, Kraemer M, Seow-Choen F, Ho YH, Eu KW. High preoperative serum carcinoembryonic antigen predicts metastatic recurrence in potentially curative colonic cancer: results of a five-year study. *Diseases of the colon and rectum* 2001 Feb;44(2): pp. 231-235.

138. Wu J, Li G, Yao Y, Wang Z, Sun W, Wang J MicroRNA-421 is a new potential diagnosis biomarker with higher sensitivity and specificity than carcinoembryonic antigen and cancer antigen 125 in gastric cancer. *Biomarkers : biochemical indicators of exposure, response, and susceptibility to chemicals* 2015 Feb;20(1): pp. 58-63.

139. Yamao T, Kai S, Kazami A et al. Tumor markers CEA, CA19-9 and CA125 in monitoring of response to systemic chemotherapy in patients with advanced gastric cancer. *Japanese journal of clinical oncology* 1999 Nov;29(11): pp. 550-555.

140. Yang AP, Liu J, Lei HY, Zhang QW, Zhao L, Yang GH. CA72-4 combined with CEA, CA125 and CAl9-9 improves the sensitivity for the early diagnosis of gastric cancer. *Clinica chimica acta; international journal of clinical chemistry* 2014 Nov 1;437: pp. 183-186.

141. Yang XQ, Chen C, Wang FB, Peng CW, Li Y. Preoperative serum carcinoembryonic antigen, carbohydrate antigen19-9 and carbohydrate antigen 125 as prognostic factors for recurrence-free survival in colorectal cancer. *Asian Pacific journal of cancer prevention : APJCP* 2011;12(5): pp. 1251-1256

142. Yang XQ, Li Y, Chen C, Peng CW, Liu SP, Liu Y. Preoperative serum carbohydrate antigen 125 level is an independent negative prognostic marker for overall survival in colorectal cancer. *Medical oncology (Northwood, London, England)* 2011 Sep;28(3): pp. 789-795.

143. Ychou M, Duffour J, Kramar A, Gourgou S, Grenier J. Clinical significance and prognostic value of CA72-4 compared with CEA and CA19-9 in patients with gastric cancer. *Disease markers* 2000;16(3-4): pp. 105-110.

144. Ye HM, Lu YZ, Liang XM et al. Clinical significance of combined testing of YKL-40 with CEA in Chinese colorectal cancer patients. *Clinical laboratory* 2014;60(3): pp. 397-405.

145. Yi JH, Kim H, Jung M et al. Prognostic factors for disease-free survival after preoperative chemotherapy followed by curative resection in patients with colorectal cancer harboring hepatic metastasis: a single-institute, retrospective analysis in Asia. *Oncology* 2013;85(5): pp. 283-289.

146. Yi Y, Li B, Sun H et al. Predictors of sensitivity to chemoradiotherapy of esophageal squamous cell carcinoma. *Tumour biology : the journal of the International Society for Oncodevelopmental Biology and Medicine* 2010 Aug;31(4): pp. 333-340.

147. Yi Y, Li B, Wang Z, Sun H Gong H, Zhang Z. CYFRA21-1 and CEA are useful markers for predicting the sensitivity to chemoradiotherapy of esophageal squamous cell

carcinoma. *Biomarkers : biochemical indicators of exposure, response, and susceptibility to chemicals* 2009 Nov;14(7): pp. 480-485.

148. Yilmaz O, Eroglu A, Dag E, Karaoglanoglu N, Yilmaz A. Serum levels of IGF-I and IGFBP-III and their relation with carcinoembryonic antigen and carbohydrate antigen 19-9 in cases of esophageal cancer. *International journal of clinical practice* 2006 Dec;60(12): pp. 1604-1608.

149. Yin XD, Yuan X, Xue JJ, Wang R, Zhang ZR, Tong JD. Clinical significance of carcinoembryonic antigen-, cytokeratin 19-, or survivin-positive circulating tumor cells in the peripheral blood of esophageal squamous cell carcinoma patients treated with radiotherapy. *Diseases of the esophagus : official journal of the International Society for Diseases of the Esophagus / I.S.D.E* 2012 Nov-Dec;25(8): pp. 750-756.

150. Zhang HQ, Wang RB, Yan HJ et al. Prognostic significance of CYFRA21-1, CEA and hemoglobin in patients with esophageal squamous cancer undergoing concurrent chemoradiotherapy. *Asian Pacific journal of cancer prevention : APJCP* 2012;13(1): pp. 199-203.

151. Zhong W, Yu Z, Zhan J et al. Association of serum levels of CEA, CA199, CA125, CYFRA21-1 and CA72-4 and disease characteristics in colorectal cancer. *Pathology oncology research : POR* 2015 Jan;21(1): pp. 83-95.
